# Safer communities; how the first forensic community mental health team helped improve mental health, battle stigma, and reduce offending

**DOI:** 10.1192/j.eurpsy.2022.1538

**Published:** 2022-09-01

**Authors:** M.H. Siddiqui, S. Reagu, M. Al Abdulla

**Affiliations:** Hamad Medical Corporation, Psychiatry Department, Doha, Qatar

**Keywords:** Qatar, forensic psychiatry, readmission, Recidivism

## Abstract

**Introduction:**

Qatar established its Forensic Community Mental Health Team (FCMHT) in 2019 as part of the region’s first comprehensive forensic psychiatry service. We present here the data on clinical and offending outcomes since its establishment and compare this with data from before the service was established

**Objectives:**

To compare clinical and offending outcomes in mental health patients with criminal offending histories in Qatar before and after the establishment of Forensic Community Mental Health Team (FCMHT).

**Methods:**

This is a retrospective study comparing the socio-demographical characteristics, clinical outcome and recidivism measures of forensic patients, under the FCMHT for the last two years with data from a similar period before the services were in place.

**Results:**

Data for 170 patients in total was analyzed. 85 patients currently under the active care of forensic community team were matched with a comparable group before the establishment of the services. The re-admission and reoffending rates after the establishment of the service over 1 year of follow up was 15% and 20% respectively compared with 60% and 85% of the group before the service.

**Conclusions:**

Since its inception, the FCMHT has made significant positive impact on quality of life, mental well-being and safety of patients under its care. Close working relationships with criminal justice system, families and carers has helped fight stigma and promote safer community.

**Disclosure:**

No significant relationships.

